# Applications of 3D printing in breast cancer management

**DOI:** 10.1186/s41205-021-00095-8

**Published:** 2021-02-09

**Authors:** Arpine Galstyan, Michael J. Bunker, Fluvio Lobo, Robert Sims, James Inziello, Jack Stubbs, Rita Mukthar, Tatiana Kelil

**Affiliations:** 1 University of California, 1600 Divisadero St, C250, Box 1667, San Francisco, CA 94115 USA; 2 Department of Radiology, Center for Advanced 3D Technologies, 1600 Divisadero St, C250, Box 1667, San Francisco, CA 94115 USA; 3 University of Florida, 3100 Technology Pkwy, Orlando, FL 32826 USA; 4 Department of Surgery, University of California, 1600 Divisadero St, C250, Box 1667, San Francisco, CA 94115 USA

## Abstract

Three-dimensional (3D) printing is a method by which two-dimensional (2D) virtual data is converted to 3D objects by depositing various raw materials into successive layers. Even though the technology was invented almost 40 years ago, a rapid expansion in medical applications of 3D printing has only been observed in the last few years. 3D printing has been applied in almost every subspecialty of medicine for pre-surgical planning, production of patient-specific surgical devices, simulation, and training. While there are multiple review articles describing utilization of 3D printing in various disciplines, there is paucity of literature addressing applications of 3D printing in breast cancer management. Herein, we review the current applications of 3D printing in breast cancer management and discuss the potential impact on future practices.

## Introduction

3D printing, also referred to as additive manufacturing and rapid prototyping, involves the creation of 3D objects from 2D virtual data using material that is either fused or deposited layer-by-layer from the ground up [1]. Segmentation software programs identify specific voxels within the anatomy of interest, isolate the region of interest into its core constituents, and generate a file that is recognizable by 3D printers. This file can then be modified through design software programs in order to create a model that is optimized for printing and possesses the characteristics desired by the provider [2]. 3D printed models are particularly beneficial for surgeons and have been utilized in almost all surgical subspecialties for pre-surgical planning, intraoperative guidance, and the productions of customized implants [1–9]. During preoperative surgical planning, the models allow surgeons to anticipate potential difficulties and tailor their surgical approach accordingly. By extension, 3D models help reduce overall intraoperative time, minimize anesthetic dosage and optimize surgical outcomes [2, 5, 9]. They are also used to facilitate interdisciplinary communication between health care providers and can enhance education of trainees and patients. This article reviews the applications of 3D printing in breast cancer management.

### Breast conservation surgery and tumor localization

Breast cancer is the most common form of non-cutaneous cancer and the second most common cause of cancer-related deaths [10]. A personalized approach to breast cancer is important as breast cancer is a heterogeneous disease where management is dependent on multiple patient-specific factors [11]. Surgical management usually includes either breast conservation surgery (BCS) or mastectomy. Although mastectomy is an important and definitive treatment option for some patients, it is often associated with substantial psychological, social, and sexual sequelae, as well as significant body image distortion [12]. BCS followed by radiation therapy has been validated as an equivalent alternative to mastectomy, with similar survival rates, acceptable rates of local recurrence, and better cosmetic outcomes [13–15]. Approximately 60–75% of American women with early stage breast cancer are treated with BCS [16]. Traditionally, patients with inadequate tumor size to breast size ratios were managed with mastectomy, however recent advances in oncoplastic techniques allow more patients with extensive disease to be considered for BCS [17].

**Fig. 1:**
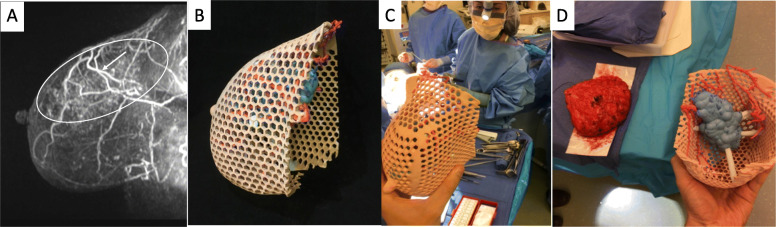
**a** Sagittal 3D maximum intensity projection (MIP) image from a contrast enhanced MRI showing extent of abnormal non –mass enhancement in the right upper breast (white ellipse) and surrounding vessels (arrow). **b** A 3D printed model derived from this breast MRI. **c** A breast surgeon using the 3D printed model intraoperatively to visualize the location of the tumor as well as its relationship to adjacent vessels. **d** Side by side comparison of the excised specimen and the 3D model

A successful BCS requires multidisciplinary communication and planning between the surgeon, radiologist, pathologist, radiation and medical oncologist. The goal is to safely remove the tumor with adequate surgical margins and provide good cosmetic outcome without compromising survival. In patients undergoing BCS, negative margin status greatly reduces risk of local recurrence and increases relapse-free survival. Wire needle localization and non-wire localization such as seeds are accepted standards methods used to guide intraoperative surgical excision of nonpalpable breast lesions. However, determination of tumor size and extent remains imprecise regardless of the method utilized for BCS [18]. This results in positive margins requiring re-excision 22–34% of the time [18–20]. In addition to causing patient anxiety and extra health care expenses, re-excision for positive margins impairs cosmetic outcomes and increases the potential for complications from surgery [21, 22]. Furthermore, localization techniques such as wires and seeds require the surgeon to estimate the 3D location and extent of cancer typically from 2D post localization mammography images, which are limited by their acquisition plane or display format. Furthermore, wires may enter the breast skin at a distance from the site of the cancer, requiring additional estimation [23]. Supplementing wire needle and non-wire localization methods with perioperative use of 3D printed models provides a tangible depiction of the patient’s breast and disease extent, facilitating planning of surgical options and approach. The physical models also provide detailed information about anatomic relationships between the tumor, overlying skin, nipple, and pectoralis muscle, beyond what is traditionally depicted with mammograms and magnetic resonance imaging (MRI), and enhance visualization of the overall breast and tumor volume (Fig. 1). This in turn aids with achievement of negative surgical margins [24].

Despite the high-resolution images obtained by MRI, a natural shift occurs in both the position and shape of the breast tumor as a patient moves from the prone position, in which the images were obtained, to the supine position intraoperatively. The lateral displacement of the breast observed intraoperatively may also alter anatomic relationships used to guide resection of large areas of disease despite the use of localization devices. With 3D printing, tumor localization can be optimized as finite information can be provided regarding tumor morphology, shape, and location. Barth et al. (2017) accurately localized tumors using a 3D-printed bra-like device that matches the breast surface when the patient is in the supine position [23]. The 3D printed bra-like device was fabricated with features that allowed the surgeons to mark the edges of the tumor on the skin surface and inject blue dye into the breast 1 cm from the tumor edges. Using this device, they were able to accurately localize 18 out of 19 cancers and achieved negative margins in all cases.

**Fig. 2:**
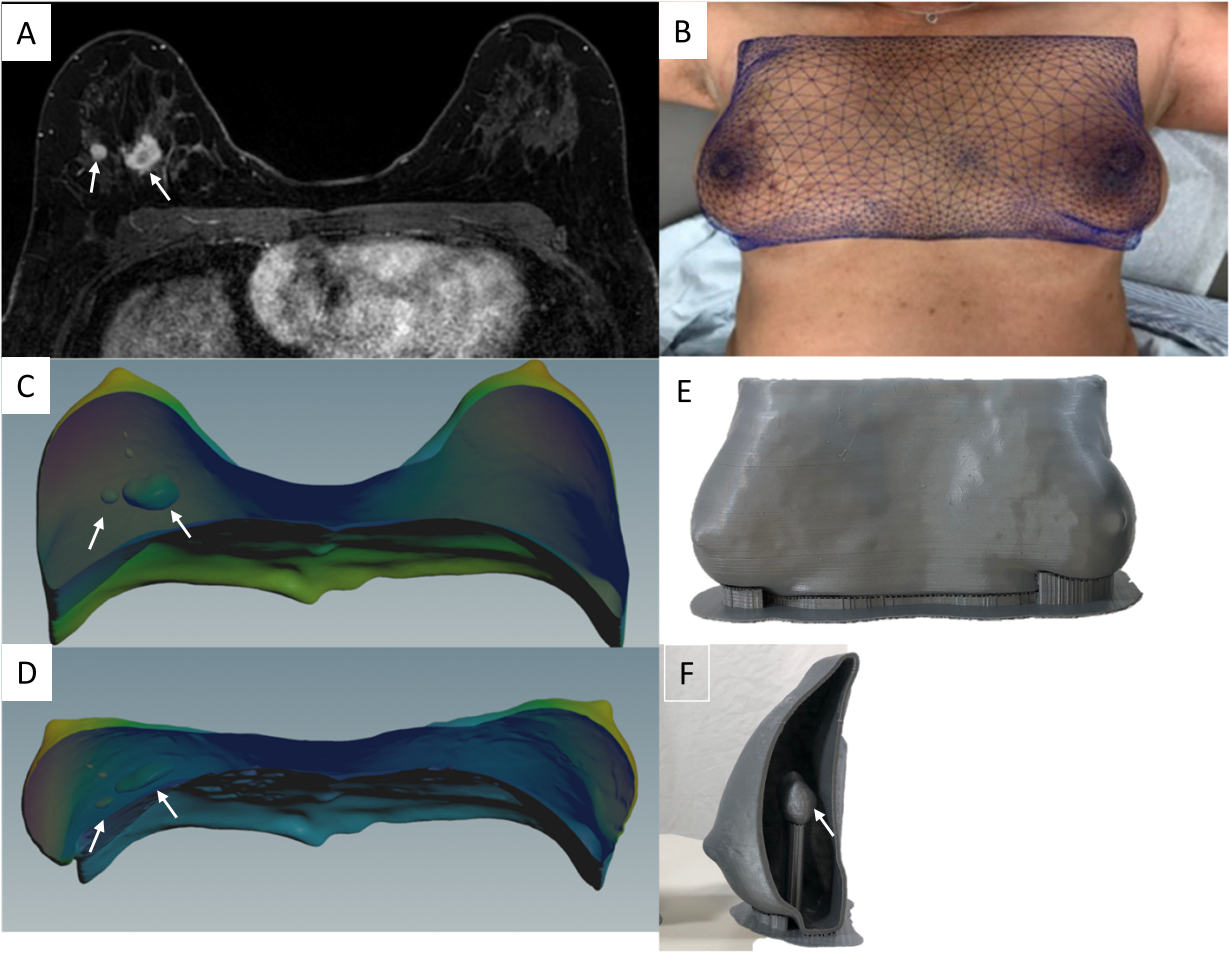
**a** Axial contrast-enhanced MRI obtained in the prone position showing two adjacent masses in the right breast (arrows). **b** A photograph from the same patient positioned supine used to create an overlying map which is then used to to warp the prone 3D virtual model (**c**) obtained from the original prone MRI dataset into a simulated 3D model in the supine position (**d**). The expected displacement and deformation of the two masses (arrows) from the prone (**c**) to the simulated supine position (**d**) is also shown. **e**, **f** 3D printed model fabricated from the estimated supine position

**Fig. 3:**
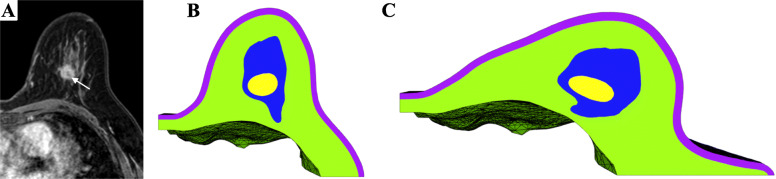
**a** Axial contrast-enhanced MRI of the left breast in prone position showing a central mass (arrow). **b** Axial cross-section of multi-compartment reconstruction in prone position. The 3D reconstruction includes skin (purple), adipose tissue (green), fibroglandular tissue (blue), and mass (yellow). **c** Simulated multi-compartment reconstruction in the supine or surgical position. The supine state was achieved by applying gravity to the model (in vivo gravity loading). The entire model, including each compartment, experience displacement and deformations proportional to their inherent mechanical properties

**Fig. 4:**
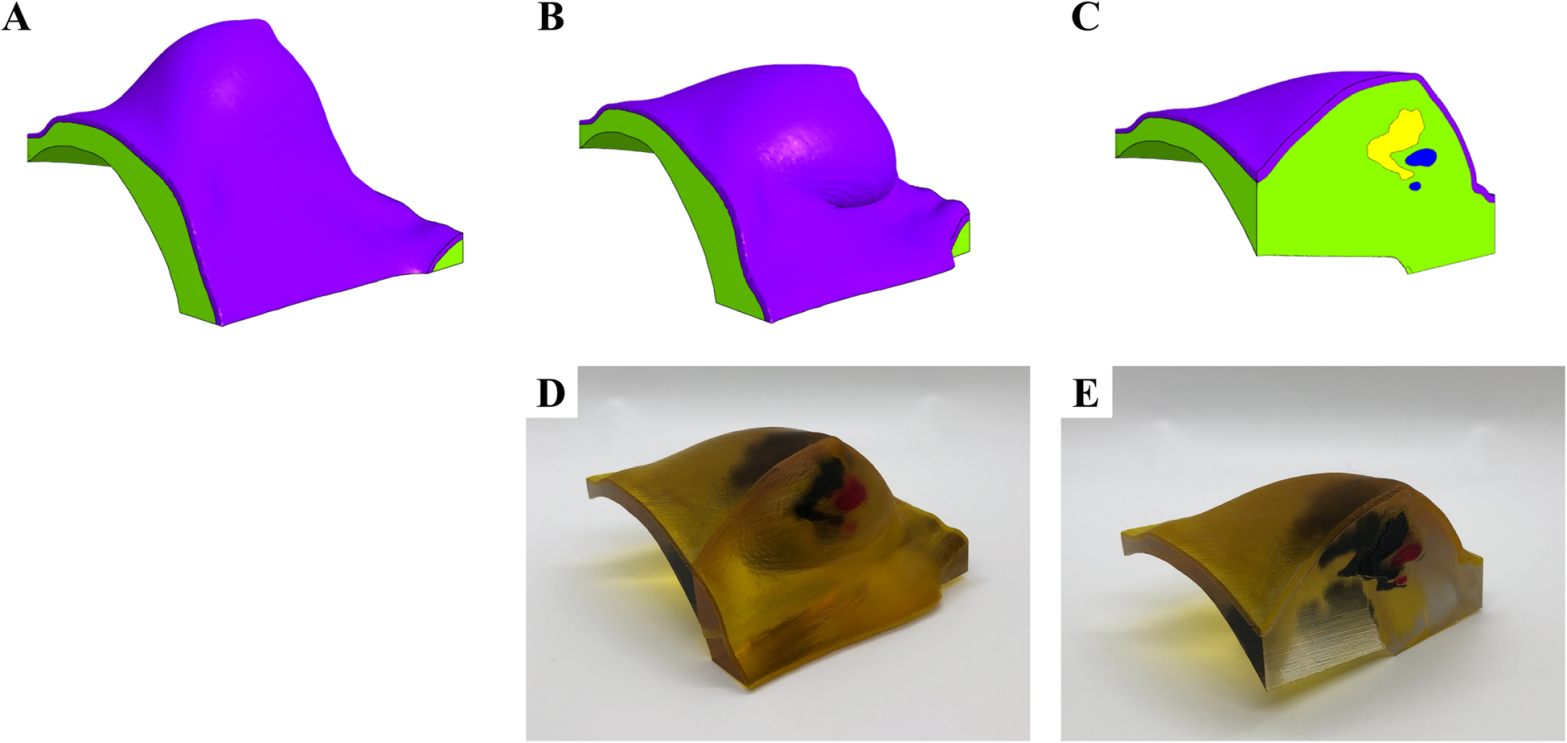
3D Printed, Patient-Specific Model of Simulated Supine Position. The 3D patient specific reconstruction of the left breast in the prone position (**a**). The simulated supine position of the 3D patient-specific reconstruction (**b**). A parasagittal cross-section of the supine model featuring fibro-glandular tissue (yellow) and two masses (blue) (**c**). The 3D printed, multi-material model of the simulated supine position, featuring the skin (translucent yellow), the fibro-glandular tissue (cyan), and the two masses (magenta) (**d**). Half of the 3D printed model (**e**), split along the parasagittal plane used in (**c**)

In most cases, with MRI typically obtained in the prone position, finite element simulations can address this problem by estimating the displacement and deformation of the breast tissue as the patient shifts from prone to supine position [25, 26].. The resulting map is then used to warp the original prone MRI dataset into a simulated supine position (Fig. 2). Alternatively, a multi-compartment finite element simulation can estimate the displacement and deformation of skin, fibro-glandular and adipose tissue as well as the changes in the location and shape of tumor from a prone to a supine position (Fig. 3). Using multi-material and multi-color 3D printers, 3D models of the estimated deformed configuration can be fabricated. These models created with varying colors and material properties can highlight all the tissue compartments, including skin, fibroglandular tissue, fibro-glandular tissue, and the breast tumor (Fig. 4).

### Breast reconstruction surgery

When BCS is not technically feasible or desired, mastectomy is recommended and is utilized in an estimated 28–60% of women with breast cancer; of those, approximately 30% subsequently undergo breast reconstruction [27]. Women who undergo breast reconstruction after mastectomy experience better psychosocial adjustment and quality of life than women who receive mastectomy without reconstruction [28]. Multiple methods of breast reconstructive surgery exist. While implant-based reconstruction is the most common form accounting for approximately 80% of breast reconstructive operations, autologous flap reconstructions have several advantages, including creation of a more natural-appearing breast and improved quality of life [29]. Women who underwent flap procedures report significantly greater satisfaction with their breasts, sexual well-being, and psychosocial adjustment than women who underwent implant reconstruction [30].

**Fig. 5:**
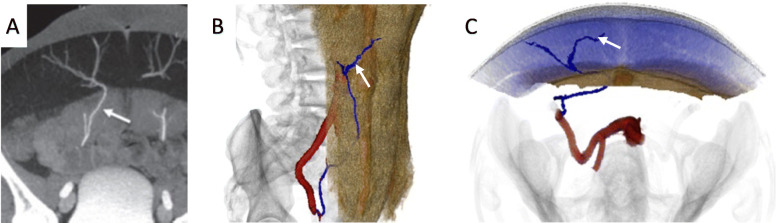
**a** Axial Maximum intensity Projection (MIP) image from a CTA demonstrating the deep inferior epigastric perforating vessels (arrow). **b** Coronal and **c** axial projections of a 3D rendering obtained from the CTA images showing the subfascial intramuscular course of the vascular tree

**Fig. 6:**
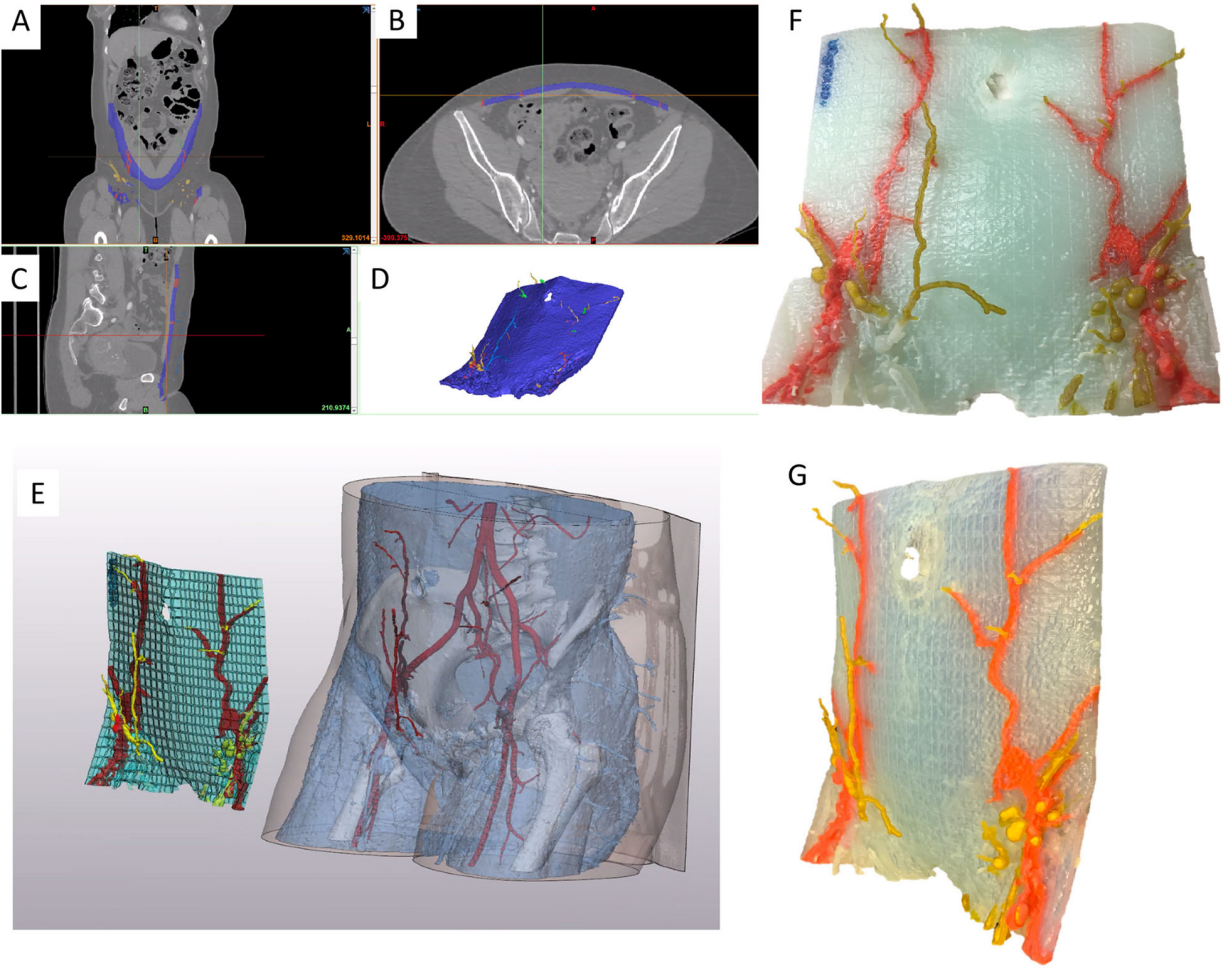
**a** Coronal, **b** axial, and **c** Sagittal images from a preoperative CTA are used to create a 3D model depicting the deep inferior epigastric vascular tree. A segmentation software (MIMICS; Materialise, Belgium) is used isolate vessels of interest and the abdominal muscle inorder to highlight the intramuscular course of these vessels (**d**, **e**). The virtual model is subsequently printed using the Stratasys Connex J735 3D printer (Eden Prairie, Minn) and is shown in the coronal (**f**) and (**g**) sagittal projection. This model can be used intraoperatively to guide dissection of vessels

Autologous breast reconstruction with deep inferior epigastric artery perforator (DIEP) flap has become an integral component of the holistic treatment of breast cancer patients. During this reconstruction, subcutaneous fat and skin from the lower abdomen are transferred as a vascularized free flap to reconstruct the breast. Yet, similar to other vascular anastomoses, autologous breast reconstructions are susceptible to microvascular anastomotic failure that threatens free flap survivability. Flap survival relies on the identification and safe harvest of suitable perforator vessels [31]. These vessels usually take a tortuous course through the rectus abdominis muscle and intramuscular dissection is often time-consuming and laden with potential unintended injury to critical vessels. Although imaging modalities such as preoperative computed tomography angiography (CTA), magnetic resonance angiography (MRA) and doppler ultrasonography have been instrumental in identifying suitable perforators, they are displayed on a 2D surface and do not adequately address the greater challenge to harvesting abdominal flaps, which is the inability to clearly conceptualize the subfascial intramuscular course of the DIEP vascular tree [32–34]. Selection of dominant vessels for microvascular anastomosis based on 2D images is challenging. Images displayed on 2D monitors provide inadequate information regarding vessel trajectory, allow for subpar manipulation of the original image, and have restricted or fixed planes of rotation that hinder the ability to view relationships of interest, which is particularly important when estimating the amount of breast tissue available for reconstruction [24]. 3D printed models have been shown to facilitate the intramuscular dissection of perforator vessels by depicting the course and trajectory of the subfascial vascular tree and allowing the surgeon to hold and view the model from various vantage points (Figs. 5 and 6) [24, 35]. The tactile feedback rendered by the models has also been shown to facilitate superior spatial understanding [36].

**Fig. 7:**
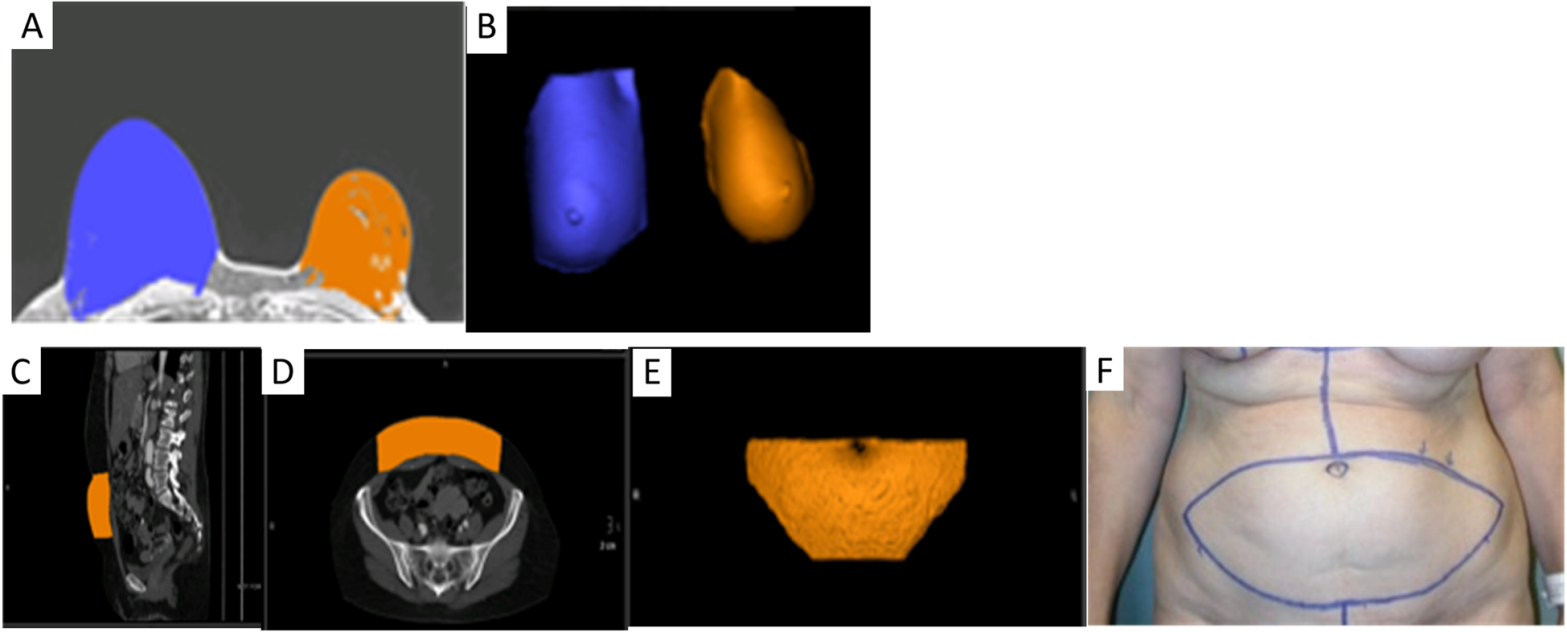
**a** Axial contrast-enhanced breast MRI and **b** 3D volume rendering obtained from this MRI used to analyze the volume of the right (blue) and left (orange) breasts, prior to planned left breast mastectomy with subsequent abdominal flap reconstruction. The volume of abdominal flap tissue that needs to be harvested to match the volume of the contralateral right breast was calculated and marked on the preoperative CTA as shown with representative sagittal (**c**) and axial (**d**) images. A virtual 3D model (**e**) was then generated which was used to mark the skin intraoperatively so that an appropriate volume of abdominal flap tissue can be harvested

Furthermore, current methods of selecting the desired volume and shape of breast implants or soft tissue flaps are inaccurate as they are subjective and dependent on the individual surgeon’s experience. Therefore, either excessive or inadequate amounts of tissue are often excised. During the preoperative period, 2D images and physical examination are used for visual estimation of the anticipated soft tissue flap or implant volume. Intraoperatively, surgeons then match the volume of the breast with the volume of the flap that is to be used. The flap is harvested and weighed after it is detached from the donor site and prior to anastomosis to the chest wall vessels. The weight of the removed breast and flap are compared and the flap is excised serially until optimal symmetry is achieved with the contralateral breast. If prolonged, these steps can increase the chances of microvascular anastomosis failure and fat necrosis. Since it is difficult to accurately estimate flap volume before excision, an excessive amount may be excised with the remaining tissue trimmed and discarded. This is particularly problematic for lean patients, for whom excessive tissue excision may increase risk of donor site repair and hypertrophic scarring [37]. Current methods of matching flap volume to desired breast size involve trial-and-error estimation and intraoperative revision of flap design. This prolonged intraoperative tissue plane alteration can lead to fat necrosis and secondary procedures may be needed to improve breast asymmetry. 3D models can be used for accurate analysis of breast volume, shape, and contour preoperatively, leading to symmetric surgical outcomes [38] (Fig. 7). Another important benefit of optimizing preoperative planning with 3D printed models is the potential to minimize the rate of fat necrosis which currently remains as high as 35% [39]. Improved understanding of the course of perforators and perfusion characteristics may be useful in reducing the risk of fat necrosis, unintended vessel injury, and the need for secondary procedures [36, 40]. Patient-specific 3D printed breast molds can also be used intraoperatively to facilitate the surgeon in shaping the contour and positioning of the autologous tissue by placing the free flap inside the mold in a manner that adapts to the shape of the template [41]. This allows matching the dimensions of the desired breast volume and shape, optimizing breast reconstruction outcomes [24].

### Physician-patient and interdisciplinary communication

**Fig. 8:**
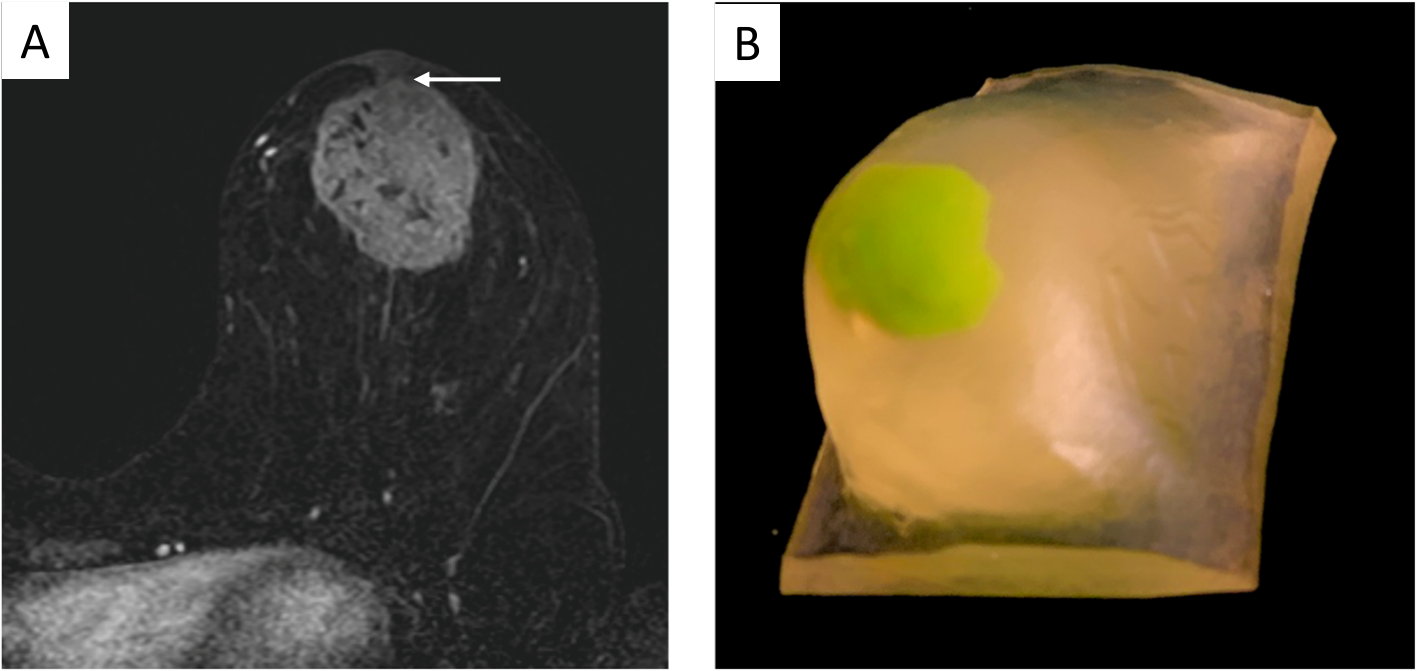
**a** Axial contrast-enhanced MRI showing a left breast mass with direct invasion of the toverlying nipple areolar complex (arrow). **b** A patient-specific 3D printed model generated from the MRI was used for patient education and obtaining of informed consent. 3D printed models allow patients to easily understand the extent of disease and rationale for a specific surgical approach

Providers often use radiologic images and/or standardized pictures to facilitate patient understanding of the nature and extent of disease. Yet, significant limitations exist with these traditional approaches. First, and most importantly, these images are 2D. In order to fully appreciate the breadth of information contained within an image, a patient must have a general understanding of how these images are produced and developed, and what limitations exist within each imaging modality. In addition, patients need a general appreciation for the anatomical structures represented within each image. Given the level of complexity, patients may have a difficult time understanding the nature and extent of their disease. 3D printed models serve as great communication tools for patients who are trying to better understand their disease and treatment options and have been shown to improve comprehension in informed consent [42, 43]. With customized 3D models, patients can better appreciate the tumor burden relative to their breast size, and make an informed decision regarding BCS vs mastectomy (Fig. 8).

In breast surgery, 3D printed models facilitate communication between the patient, breast surgeon and plastic surgeon when considering BCS with oncoplastic reconstruction. The patient specific 3D printed models can be used in discussion between patient-surgeon, surgeon-radiologist, and surgeon-pathologist possibly impacting consent, preoperative planning, and assessment of pathologic concordance [24]. By improving tools used to educate patients on their disease, patients can make more informed decisions that will ostensibly promote better treatment decisions which in turn improve patient satisfaction rates, help reduce the need for secondary surgeries, and improve quality of life measures.

### Education and simulation

The application of 3D printing is becoming increasingly adopted in training and simulation of complex surgical and image guided procedures. Traditionally, surgical training has involved direct work on cadavers or the “see one, do one, teach one” approach. Although cadavers are anatomically accurate, they are too-expensive, do not retain live-tissue characteristics, lack the appropriate pathology, and are limited in supply [1, 44]. Training on 3D printed models can be done virtually anywhere, avoiding the cost and complexity of operating in the controlled environments required for animals and human cadavers. 3D printed models are particularly of great benefit for novice practitioners in training by supplementing early operating experiences with a low-risk environment in which the trainees can learn and perfect their skills before they are allowed to work on patients [45, 46].

**Fig. 9:**
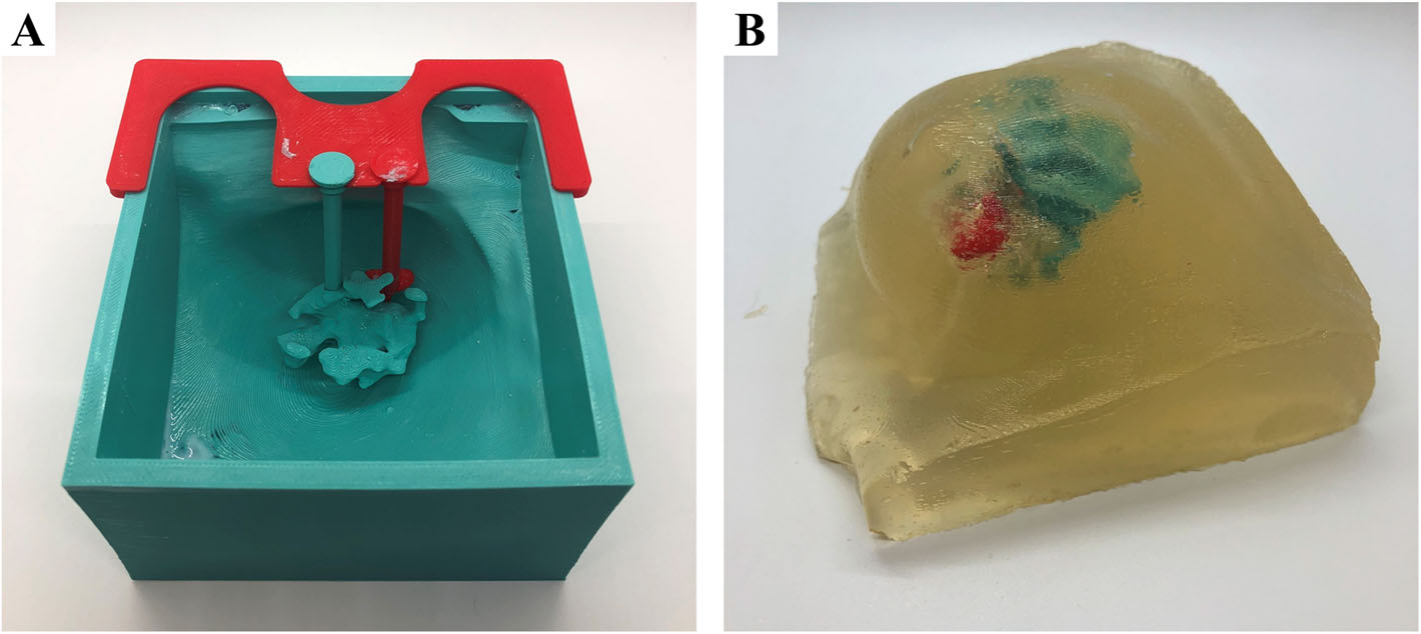
Patient-specific molds were generated by subtracting a patient’s 3D breast model from an enclosing volume (**a**). Internal structures, such as fibroglandular tissue and masses, were held in place with custom fixtures designed onto the mold (**a**). The mold, internal structures and fixtures were 3D printed using polylactic acid (PLA), a common and inexpensive 3D printing material (**a**). Silicone was poured into the 3D printed mold (**b**). Upon setting, fixtures holding internal structures were removed prior to releasing the mold. The cast was removed from the mold by gently separating the edges and tapping on the bottom of the mold (**b**)

Similarly, 3D printed breast phantoms aid in the teaching and training of ultrasound-guided core needle biopsy techniques [47]. Although chicken breast has been traditionally used for training of ultrasound guided needle biopsy techniques, it is non-reusable, environmentally unfriendly, and unsanitary risking contamination of biopsy instruments which leads to increased waste and cost. Phantoms made of gelatin provide low-cost alternative, however tend to be too fragile with limited shelf-life and reusability. 3D printed teaching models not only serve as a more cost-effective alternative, but the versatility and customizability of 3D printing can also be used to generate an expansive library of anatomical variation in breast sizes, densities and pathologies (Fig. 9).

### Quality control

Physical phantoms are commonly used as surrogates of breast tissue to evaluate performance of breast imaging systems. However, most traditional phantoms do not reproduce the anatomic heterogeneity of real breast tissue. 3D printing creates the opportunity to fabricate more complex and anatomically accurate breast phantoms that can be used for quality assurance testing as well as development and optimization of breast imaging systems. Since 3D printed phantoms are reproducible, customizable, and cost-efficient, a collection of representative patient models can be fabricated to evaluate the effect of anatomic variability on system performance [48].

Currently, the majority of phantoms are designed for use with a single imaging modality, thus multiple phantoms are required for different imaging systems. 3D printing overcomes this limitation by enabling the construction of multi-purpose customizable breast phantoms made of tissue-equivalent materials that are compatible with multiple imaging modalities [49]. These phantoms can also be embedded with various inserts for simulating different breast pathologies such as masses and micro-calcification [50]. Ikejimba et al. (2017) generated breast models using a 3D printer that used relatively inexpensive materials with attenuation properties similar to that of real-life breast tissue and lesions of varying sizes and physical characteristics. The resulting 3D printed phantoms and their respective mammographic and tomosynthesis images demonstrated breast backgrounds comparable to that of normal fibroglandular tissue, while still demonstrating a wide range of pathologies [51].

## Future directions

### Personalized radiation therapy

3D printing is promising to aid in delivery of personalized radiation therapy. A bolus is an artificial object placed over the treatment area to modify radiation dose and shields are used to protect adjacent structures not intended to be exposed to radiation. 3D printing can be used to design customized patient specific boluses and shields to allow homogeneous distribution of radiation dose to the area of interest while sparing adjacent normal tissue [52]. Another application of 3D printing is in brachytherapy where a radiation source is implanted next to the area requiring treatment. The traditional standardized implants which are currently used do not conform to patients’ specific anatomy and precise positioning is often challenging. These implants are also prone to shifting during movement resulting in suboptimal dose to the target and unwanted exposure to adjacent organs. 3D printed customized brachytherapy templates provide a much better fit thereby increasing patient comfort and reducing shifts due to movement [53]. Customized implants with curved internal channels can also be used to reach targets that may not be accessible with existing standardized implants [53]. In high-dose-rate brachytherapy, the number and positions of the catheters are traditionally chosen manually using radiation planning CT or ultrasound. 3D printing allows a simple, fast, and efficient method for real time brachytherapy treatment which utilizes a reduced number of catheters than the traditional approach [54, 55].

### Bioprinting

Bioprinting is an extension of traditional 3D printing processes, where biomaterials such as cells and growth factors are used to create tissue-like structures that mimic natural tissues such as skin and blood vessels. This novel technology is promising to address challenges encountered with current breast reconstruction techniques [56]. For instance, 3D bioprinting can be used to generate a biodegradable scaffold that can be combined with autologous adipose tissue in lipofilling. Lipofilling is a reconstructive and aesthetic technique whereby autologous fat is used for filling defects and remodeling body contours [57]. In breast cancer surgery, lipofilling can be used to correct defects following wide local excision, improve symmetry after lumpectomy, replace volume of implants in unsatisfactory breast reconstruction outcomes, and even achieve whole breast reconstruction following mastectomy with serial fat grafting [58]. Current techniques have several drawbacks including high resorption rate of injected fat and fat necrosis due to lack of vascularization. Bioprinting allows fabrication of patient-specific bio-absorbable scaffolds that can be seeded with various stem cells and growth factors, closely resembling the extracellular matrix that can support the generation of blood vessels [59–61]. These scaffolds which subsequently get resorbed by the body, safely contain the injected fatty tissue and minimize the significant volume loss of breast fat usually observed in lipofilling.

Another important promising application of bioprinting is the recreation of the nipple-areola complex (NAC) during breast reconstruction [62]. NAC is highly correlated with patient satisfaction and body image perception after breast reconstruction, however current techniques such as local flaps and pigmented skin grafts have unpredictable long-term outcomes [63]. Using adipose-derived stem cells, functional, durable, and patient-specific tissue constructs can be generated that closely mimic physiologic tissue, have multipotent differentiation capacity, and react to normal tissue-specific development cues. Bioprinting is promising a novel approach to generate replacement nipple tissue, though much work remains to be done in this area. In addition to tissue replacements for breast reconstruction, bioprinting can also be used to engineer breast tissue models that serve as valuable tools in cancer research and drug screening applications [64, 65].

## Conclusion

3D printing is poised to revolutionize breast cancer surgery by allowing patient-specific pre-surgical planning and customized intraoperative surgical guides for breast conservation and reconstruction. The enhanced understanding of anatomic relationships rendered by 3D models has allowed better esthetic surgical outcomes while simultaneously achieving negative surgical margins. In addition, 3D models serve as great teaching tools for patients and trainees and enhance interdisciplinary communication between various health care providers. 3D printed phantoms are proving to be superior to traditional phantoms that are used for quality assurance of breast imaging systems. Bioprinting and personalized radiation therapy are emerging fields which are promising to address challenges encountered with current breast cancer management approaches.

## Data Availability

Not applicable.
