# Correction to: Applications of 3D printing in breast cancer management

**DOI:** 10.1186/s41205-021-00109-5

**Published:** 2021-07-07

**Authors:** Arpine Galstyan, Michael J. Bunker, Fluvio Lobo, Robert Sims, James Inziello, Jack Stubbs, Rita Mukhtar, Tatiana Kelil

**Affiliations:** 1 University of California, 1600 Divisadero St, C250, Box 1667, San Francisco, CA 94115 USA; 2 Department of Radiology, Center for Advanced 3D Technologies, 1600 Divisadero St, C250, Box 1667, San Francisco, CA 94115 USA; 3 University of Florida, 3100 Technology Pkwy, Orlando, FL 32826 USA; 4 Department of Surgery, University of California, 1600 Divisadero St, C250, Box 1667, San Francisco, CA 94115 USA

**Correction to: 3D Print Med 7, 6 (2021)**

**https://doi.org/10.1186/s41205-021-00095-8**

Following publication of the original article [1], the author noticed that the seventh author’s family name was incorrectly spelled as “Mukthar”.

The correct spelling of the family name “Mukhtar” has been provided in this Correction.

The original article [1] has been updated.
